# Proteome profiling of polyomavirus nuclear replication centers using iPOND

**DOI:** 10.1128/jvi.00790-24

**Published:** 2024-10-31

**Authors:** Kimberly D. Erickson, Erika S. Langsfeld, Alexandra Holland, Christopher C. Ebmeier, Robert L. Garcea

**Affiliations:** 1 The BioFrontiers Institute, University of Colorado Boulder, Boulder, Colorado, USA; 2 Department of Biochemistry, University of Colorado Boulder, Boulder, Colorado, USA; 3 Department of Molecular, Cellular, and Developmental Biology, University of Colorado Boulder, Boulder, Colorado, USA; International Centre for Genetic Engineering and Biotechnology, Trieste, Italy

**Keywords:** polyomavirus, viral replication, iPOND, DNA tumor virus, proteomics

## Abstract

Polyomaviruses (PyVs) cause diverse diseases in a variety of mammalian hosts. During the life cycle, PyVs recruit nuclear host factors to viral genomes to facilitate replication and transcription. While host factors involved in DNA replication, DNA damage sensing and repair, and cell cycle regulation have been observed to bind PyV DNA, the complete set of viral and host proteins comprising the PyV replisome remains incompletely characterized. Here, the iPOND-MS technique (Isolation of Proteins on Nascent DNA coupled with Mass Spectrometry) was used to identify the proteome bound to murine PyV (MuPyV) DNA immediately following synthesis and 2 hours post-synthesis. Several novel MuPyV DNA interactors were identified on newly synthesized viral DNA (vDNA), including MCM complex members, DNA primase, DNA polymerase alpha, DNA ligase, and replication factor C. Though displaying partial overlap, the host and viral proteins bound to MuPyV DNA 2 hours post-synthesis lacked many of the replication proteins found on newly synthesized vDNA. These data help distinguish between the host factors critical for MuPyV DNA replication and those involved in downstream processing.

**IMPORTANCE:**

Polyomaviruses are the causative agents of serious diseases in humans, including progressive multifocal leukoencephalopathy (PML), BK virus nephropathy, and Merkel cell carcinoma. The exact mechanisms by which the virus replicates, and which host cell proteins are required, are incompletely characterized. Identifying the host proteins necessary for efficient viral replication in the cell may reveal targets for downstream targets that may suppress viral replication *in vivo*.

## INTRODUCTION

Polyomaviruses (PyVs) are non-enveloped, double-stranded DNA viruses replicating in the infected cell nucleus. Due to the small size of their genomes (~5 kb), PyVs rely on multiple interactions between viral and host proteins to facilitate productive infection. In addition to three capsid proteins, murine polyomavirus (MuPyV) genomes encode three “tumor” or T antigen (TAg) proteins that mediate viral DNA (vDNA) replication and virion assembly. Large T antigen (LTAg) is a multifunctional protein that is indispensable for viral genome replication. In addition to its function as a DNA helicase which binds and unwinds the origin of replication, LTAg directly binds and regulates the activity of p53 and Rb. LTAg binding to Rb results in the transcription of E2F responsive genes and stimulation of the cell cycle, while binding to p53 results in suppression of its transcriptional and pro-apoptotic activities. Middle T antigen (MTAg) associates with Src family tyrosine kinases at the cellular membrane, stimulating a complex signaling cascade contributing to host cell transformation (1). Small T antigen (STAg) binds PP2A, modulating its activity by displacing the regulatory B subunit which affects several downstream signaling pathways including cell cycle progression to the S phase and facilitation of vDNA replication (2–5). Polyomavirus STAg may facilitate replication not only through its interaction with PP2A but also by recruitment of DNA damage and repair (DDR) family proteins to replicating genomes (2, 3).

Early in MuPyV infection, many cellular proteins are recruited to nuclear subdomains termed viral replication centers (VRCs), where LTAg drives the replication of vDNA as minichromosomes (3–8). These VRCs expand and merge over the course of infection concomitantly with the assembly of virions, eventually resulting in cell lysis and virus release (1, 2). Host proteins previously shown to be recruited to MuPyV VRCs include members of the DDR pathway, which appear critical for genome replication of several viruses (3). The identification of these proteins was based upon a candidate approach, limiting a more comprehensive identification of the host proteins comprising the viral replisome.

**Fig 1:**
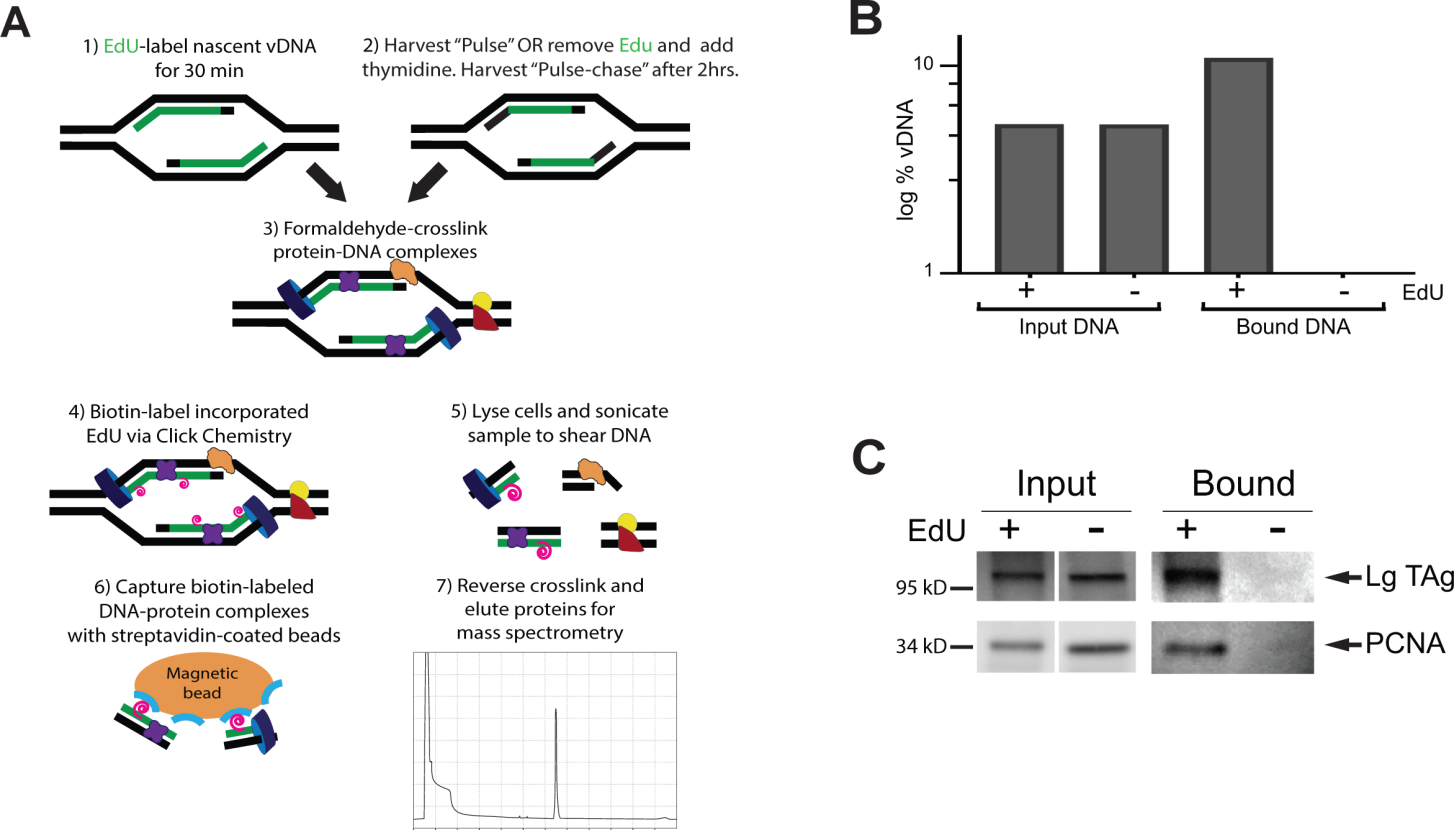
Overview of iPOND technique used to identify proteins associated with polyomavirus viral replication centers. (**A**) Schematic representation of the iPOND method. (**B**) Quantitative PCR of vDNA bound to streptavidin beads. Aliquots were removed from samples either before (Input) or after (Bound) incubation with streptavidin beads. The ratio of vDNA to total DNA was calculated and is presented as the log % vDNA. Values for “Bound DNA” in the absence of EdU (−) were too low to be displayed on this graph. (**C**) Western blot of iPOND samples using LTAg and PCNA. Aliquots were removed from samples either before (Input) or after (Bound) incubation with streptavidin beads. Aliquots for “Bound” samples were removed after reversing the formaldehyde crosslink with boiling in the presence of SDS. Input, 0.5% of total lysate; Bound, 5% of total lysate.

The iPOND-MS technique (Isolation of Proteins on Nascent DNA coupled with Mass Spectrometry) has been used to characterize proteins associated with the replisomes of DNA viruses such as adenovirus, herpes simplex virus, and vaccinia virus (4–6). iPOND-MS utilizes incorporation of the thymidine analog 5-ethynyl-2′-deoxyuridine (EdU) into replicating DNA, followed by formaldehyde crosslinking of protein-DNA complexes, bead-capture of labeled DNA-protein complexes, and mass spectrometry analysis of captured proteins (Fig. 1A) (6, 7). Thus, the iPOND technique provides a means to time-stamp proteins associated with replicating DNA at the time of the EdU pulse. These studies have revealed both common features and differences between the proteins recruited to the genomes of DNA viruses, as well as identified previously unknown cellular proteins associated with virus replication (8). In this study, we used the iPOND-MS method to identify a comprehensive repertoire of host proteins associated with the MuPyV replisome, in which EdU pulse and pulse-chase analyses were used to discriminate vDNA-bound proteins at early and later stages of replication. Immunofluorescence analysis showed localization of candidate proteins to VRCs, identified as LTAg-positive foci within infected nuclei, supporting their presence at sites of MuPyV replication. These data add to a growing body of literature describing the complex protein dynamics involved during MuPyV replication and viral genome maturation.

## RESULTS

### iPOND captures the MuPyV replisome

Our laboratory has shown previously that EdU labels any actively replicating DNA, including host and vDNA within PyV replication centers (8–11). To identify VRC proteins that associate with replicating MuPyV genomes, newly synthesized DNA in infected cells was labeled at 28 hpi using the EdU thymidine analog or with dimethyl sulfoxide (DMSO; no EdU control) (Fig. 1A) (7). The protein complexes associated with nascent vDNA (Pulse) or 2 hours post-synthesis (Pulse-Chase) were analyzed using iPOND-MS (12–14).

To determine whether vDNA was enriched in the EdU-labeled samples, aliquots of sonicated lysate were removed before or after incubation with streptavidin beads and qPCR was performed for the MuPyV VP1 coding region (Fig. 1B). While the amount of vDNA was comparable between the EdU and DMSO input samples, enrichment of vDNA was observed in the bound sample compared to the input, confirming that the majority of the captured EdU-labeled DNA was viral (Fig. 1B). As an additional validation step prior mass spectrometry (MS) analysis, the samples were analyzed by Western blot (Fig. 1C) to determine whether known viral and host DNA interactors [LTAg and the DNA-binding proliferating cell nuclear antigen (PCNA), respectively] could be recovered. Significant enrichment was observed for both LTAg and PCNA in the EdU-labeled bound sample over the DMSO control.

### iPOND recovers novel MuPyV genome interacting proteins

Pulse and Pulse-Chase sample proteomes eluted from streptavidin-coated beads were analyzed by label-free MS. The raw data were analyzed with a custom software pipeline utilizing limma (15) and MaxQuant (16) to identify significantly enriched proteins present on nascent DNA. The complete list of proteins identified in EdU and DMSO samples, as well as the relative log_2_ fold change (LFC) and *P* values calculated for each condition (limma output), is provided in Table S1. For both Pulse and Pulse-Chase EdU sample sets, proteins were considered significantly enriched relative to the DMSO control if the LFC exceeded two and the adjusted *P* value was ≤ 0.05. From the entire set of proteins identified in the EdU and DMSO samples (*n* = 5 replicates for each condition), 1,774 candidate proteins identified in the Pulse samples were retained after filtering for those proteins that had numerical (non-NaN) intensity values in at least three replicates in either the EdU or DMSO condition. Of these, 59 proteins met the cutoff of LFC exceeding two and adjusted *P* value ≤ 0.05 and were thus considered significantly enriched in the EdU samples relative to the DMSO controls.

**Fig 2:**
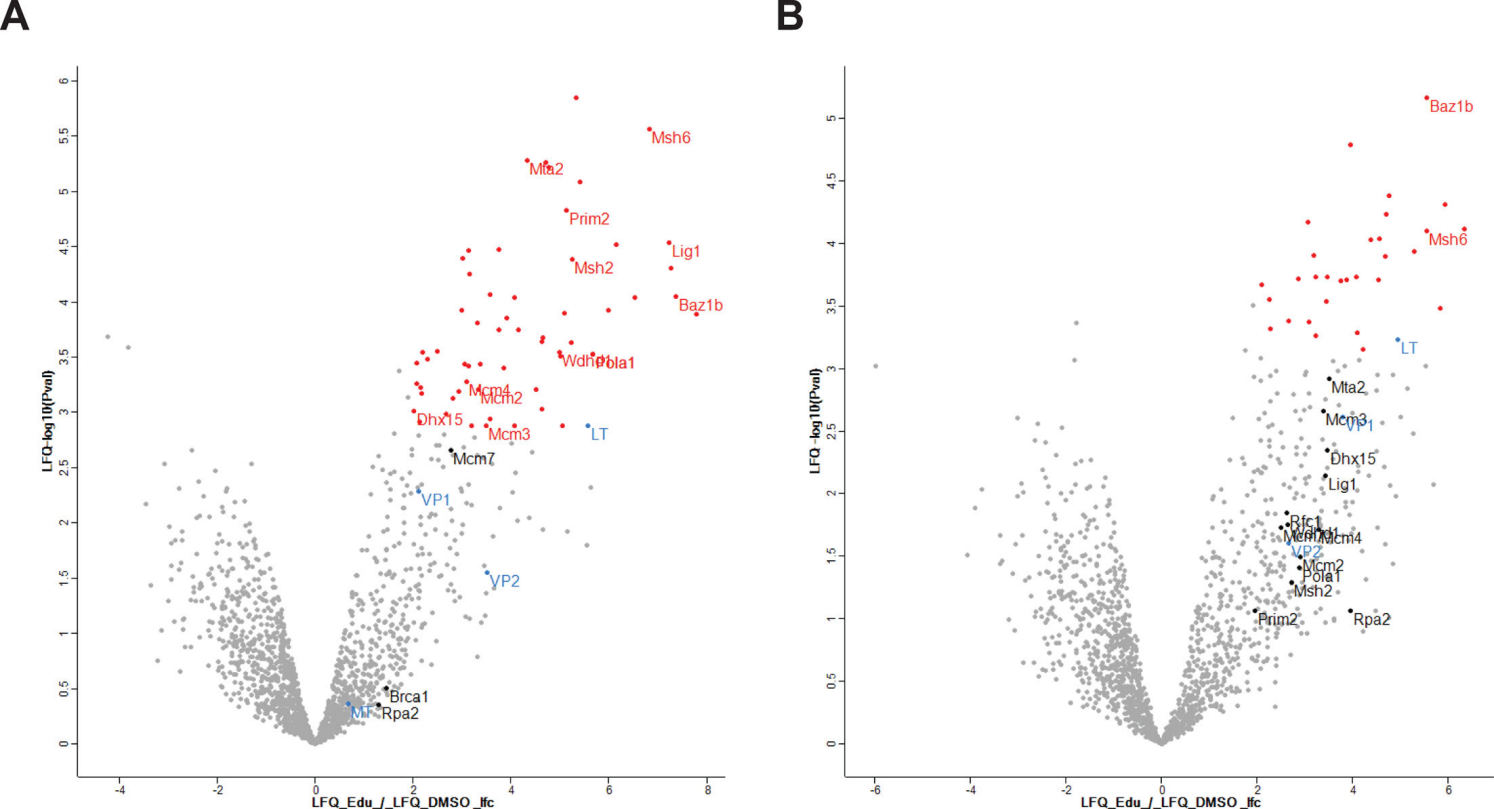
Volcano plots of iPOND candidates identified by mass spectrometry. Volcano plots show the log2 fold change in protein intensity between +EdU and −EdU samples (x-axis) plotted against the significance (y-axis) for the Pulse (**A**) and Pulse-Chase (**B**) conditions. Red dots indicate proteins with an adjusted *P* value < 0.05 and log_2_ fold change >2. Proteins of interest are labeled with gene names, and viral proteins are labeled in blue.

In Pulse-Chase samples (*n* = 5 replicated for each condition), 1,649 candidates were identified after filtering for proteins that had numerical (non-NaN) intensity values in at least three replicates in either the EdU or DMSO condition, and 31 of these were significantly enriched relative to the DMSO controls. Volcano plots were generated using MaxQuant Perseus to show the LFC between the mean protein intensities for the EdU and DMSO samples as a function of the *P* value for each protein identified in both conditions (Fig. 2A and B). Importantly, some of the most highly enriched proteins in the EdU Pulse samples included proteins that have previously been found localized to VRCs such as LTAg, PCNA, Mre11, and RPA (Fig. 2A; Table S1) (1, 8, 9). These data suggest that the iPOND method recovered both known and novel components of the MuPyV replisome. Proteins that were identified in the EdU samples but not in the DMSO control samples could not be included in the volcano plot analysis since the LFC calculation depends on numerical numerator (mean EdU intensity) and denominator (mean DMSO intensity) values. These proteins are listed in Table S2, due to the high likelihood of their biological significance.

### The proteome bound to MuPyV genomes changes as vDNA undergoes processing

**Fig 3:**
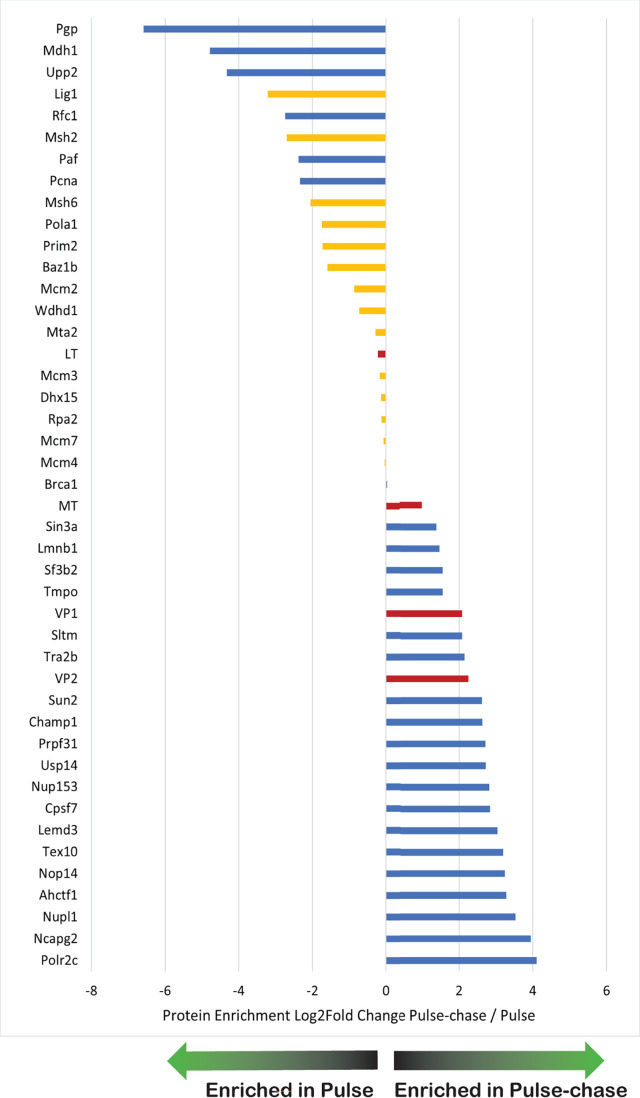
Comparison of protein enrichment on nascent DNA between Pulse and Pulse-Chase conditions. Graph showing the log_2_ fold change in protein intensity between Pulse-Chase and Pulse samples (x-axis), with corresponding gene names on the y-axis. Proteins of interest are labeled in yellow and viral proteins are labeled in red.

**Fig 4:**
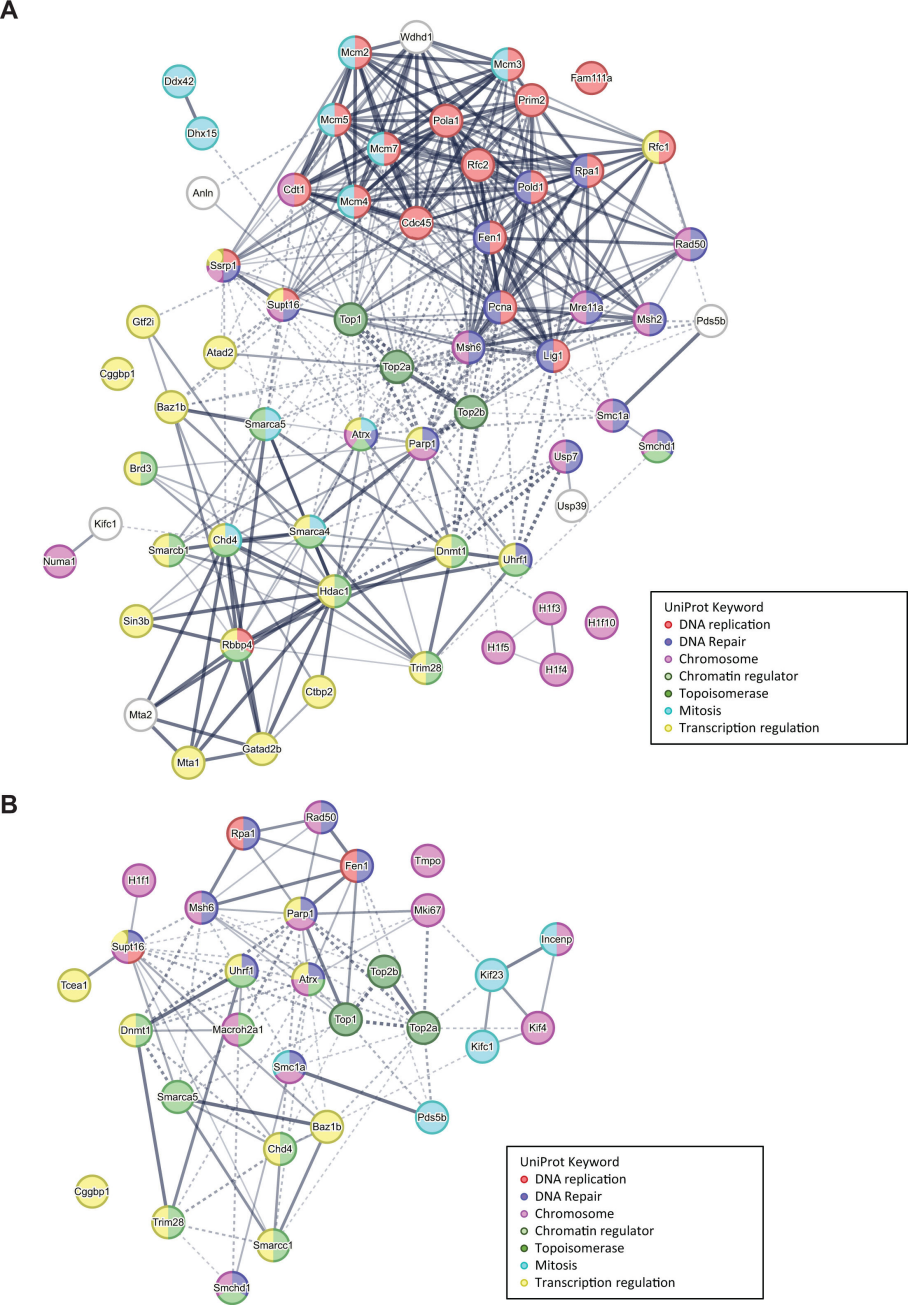
STRING cluster analysis of proteins retrieved by iPOND. Significantly enriched proteins of interest as defined in Fig. 2 and Table 1 were analyzed with the STRING database of functional associations using a medium confidence score (0.400) and a Markov Cluster Algorithm (MCL) clustering parameter of 3 (interaction sources were restricted to text-mining, experiments, and databases). Functional annotation categories are noted with distinct colors according to the legend.

Using iPOND, a novel subset of cellular proteins was identified that had not been reported for the PyV replisome (Table S1). The set of enriched proteins in the Pulse-Chase samples differed from that of the Pulse samples (Table S1; Fig. 2). To directly compare the vDNA-bound proteome in the Pulse and Pulse-Chase samples, proteins recovered from EdU-labeled vDNA for both conditions were analyzed in the same manner that the EdU and DMSO data sets were compared above (Table S3). A total of 1,176 proteins remained after filtering. As in the previous analysis, proteins not detected in either the Pulse or Pulse-Chase condition could not be compared but are likely biologically relevant (Table S4). Proteins enriched in the Pulse-Chase compared to Pulse samples (*P* value ≤ 0.01) are shown with their associated LFC (Fig. 3). We used three different analysis methods to assess whether proteins representing relevant biological processes were differentially enriched between Pulse and Pulse-Chase conditions (including EdU-only data sets). First, STRING analysis of the significantly enriched proteins illustrated the diversity between the Pulse and Pulse-Chase data sets (Fig. 4). While the proteome associated with newly synthesized vDNA was comprised of DNA replication factors, as well as factors involved in repair, chromosome organization, and transcription regulation, the proteome associated with vDNA 2 hours post-synthesis does not share many of the same DNA replication factors (Fig. 4). Second, Gene Ontology Cellular Component (GOCC) annotation was used to exclude cytoplasmic proteins from the data set and Gene Ontology Biological Process (GOBP) names were added to examine process enrichment between the Pulse and Pulse-Chase samples (17, 18). A total of 848 proteins remained after filtering by GOCC annotation to retain only nuclear proteins. A 1D annotation enrichment analysis of the resultant matrix demonstrated significant enrichment of proteins associated with DNA repair proteins in the Pulse samples when compared to the Pulse-Chase samples, and proteins associated with mRNA processing were enriched in Pulse-Chase samples when compared to the Pulse samples (Fig. S1). Third, using Kyoto Encyclopedia of Genes and Genomes (KEGG) (19) annotation instead of GOBP, a significant enrichment of proteins associated with DNA replication and mismatch repair was found in the Pulse samples as compared to the Pulse-Chase samples (Table S5). Taken together, these results support a temporal model for MuPyV replication wherein vDNA undergoes processing steps during which it associates with a different assembly of host proteins that may have distinct functions in the viral life cycle (8).

**TABLE 1:** Host and viral proteins found enriched on nascent (Pulse) and maturing (Pulse-Chase) vDNA

iPOND candidates				Biological function				
Gene name	Protein name	Enriched in Pulse	Enriched in Pulse-chase	DNA replication	DNA repair	Chromatin organization	Transcription/ RNA processing	Validated by immunofluorescence^*a*^
Anln	Actin-binding protein anillin	X						
Atad2	ATPase family AAA domain-containing protein 2	X					X	
Atrx	Transcriptional regulator ATRX	X	X			X	X	
Baz1b	Tyrosine-protein kinase BAZ1B	X	X	X	X	X	X	X
Brd3	Bromodomain-containing protein 3	X				X	X	
Brca1	Breast Cancer Type 1 Susceptibility Protein				X		X	X
Cggbp1	CGG triplet repeat-binding protein 1	X	X				X	
Chd4	Chromodomain-helicase-DNA-binding protein 4	X	X		X	X		
Ctbp2	C-terminal-binding protein 2	X				X		
Ddx42	ATP-dependent RNA helicase DDX42	X					X	
Dhx15	Pre-mRNA-splicing factor ATP-dependent RNA helicase DHX15	X					X	X
Dnajc9	DnaJ homolog subfamily C member 9	X				X		
Dnmt1	DNA (cytosine-5)-methyltransferase 1	X	X			X	X	
Fam111a	FAM111 Trypsin Like Peptidase A	X		X				
Fen1	Flap endonuclease 1	X	X	X	X	X		
Gatad2b	Transcriptional repressor p66-beta	X				X		
Gtf2i	General transcription factor II-I	X					X	
H1fx	H1.10 Linker Histone	X				X		
H2afy	Core histone macro-H2A.1		X			X		
Hdac1	Histone deacetylase 1	X				X		
Hist1h1a	Histone H1.1		X			X		
Hist1h1b	Histone H1.5	X				X		
Hist1h1d	Histone H1.3	X				X		
Hist1h1e	Histone H1.4	X				X		
Incenp	Inner centromere protein		X			X		
Kif23	Kinesin-like protein KIF23		X			X		
Kif4	Chromosome-associated kinesin KIF4		X			X		
Kifc1	Kinesin-like protein KIFC1	X	X					
Lig1	DNA ligase;DNA ligase 1	X		X	X			X
LTAg	Polyomavirus large T antigen	X	X	X				X
Mcm2	DNA replication licensing factor MCM2	X		X				X
Mcm3	DNA replication licensing factor MCM3	X		X				X
Mcm4	DNA replication licensing factor MCM4	X		X				X
Mcm5	DNA replication licensing factor MCM5	X		X				
Mcm7	DNA replication licensing factor MCM7			X				X
Mki67	Marker of proliferation Ki-67		X			X		
Mre11a	Double-strand break repair protein MRE11A	X			X			
Msh2	DNA mismatch repair protein Msh2	X			X			X
Msh6	DNA mismatch repair protein Msh6	X	X		X			X
Mta1	Metastasis-associated protein MTA1	X				X	X	
Mta2	Metastasis-associated protein MTA2	X				X	X	X
Numa1	Nuclear mitotic apparatus protein 1	X				X		
Parp1	Poly [ADP-ribose] polymerase 1	X	X		X			
Pcna	Proliferating cell nuclear antigen	X		X	X			
Pds5b	PDS5 cohesin-associated factor B	X	X	X	X	X		
Pola1	DNA polymerase alpha catalytic subunit	X		X				X
Pold1	DNA polymerase delta catalytic subunit	X		X	X			
Prim2	DNA primase large subunit	X		X				X
Rad50	DNA repair protein RAD50	X	X		X			
Rbbp4	Histone-binding protein RBBP4	X				X		
Rfc1	Replication factor C subunit 1	X		X	X			X
Rfc2	Replication factor C subunit 2	X		X	X			
Rpa1	Replication protein A 70 kDa DNA-binding subunit	X	X	X	X			
Rpa2	Replication protein A 32 KDa subunit			X	X			X
Smarca4	SWI/SNF-related, matrix-associated, actin- dependent regulator of chromatin, subfamily A, member 4	X				X	X	
Smarca5	SWI/SNF-related, matrix-associated, actin-dependent regulator of chromatin subfamily A member 5	X	X			X	X	
Smarcc1	SWI/SNF complex subunit SMARCC1		X			X	X	
Smc1a	Structural maintenance of chromosomes 1A	X	X		X	X		
Smchd1	Structural maintenance of chromosomes flexible hinge domain-containing protein 1	X	X		X	X		
Ssrp1	FACT complex subunit SSRP1	X				X	X	
Supt16	FACT complex subunit SPT16	X	X			X	X	
Tcea1	Transcription elongation factor A protein 1		X				X	
Tmpo	Thymopoietin		X	X		X		
Top1	DNA topoisomerase 1	X	X	X	X	X	X	
Top2a	DNA topoisomerase 2-alpha	X	X	X	X	X	X	
Top2b	DNA topoisomerase 2-beta	X	X	X	X	X	X	
Trim28	Transcription intermediary factor 1-beta	X	X			X	X	
Uhrf1	E3 ubiquitin-protein ligase UHRF1	X	X		X	X	X	
Usp39	U4/U6.U5 tri-snRNP-associated protein 2	X			X		X	
Usp7	Ubiquitin-specific peptidase 7	X			X	X		
Wdhd1	WD repeat and HMG-box DNA-binding protein 1	X		X				X

^*a*^ In this study.

### iPOND protein candidates are associated with VRCs

**Fig 5:**
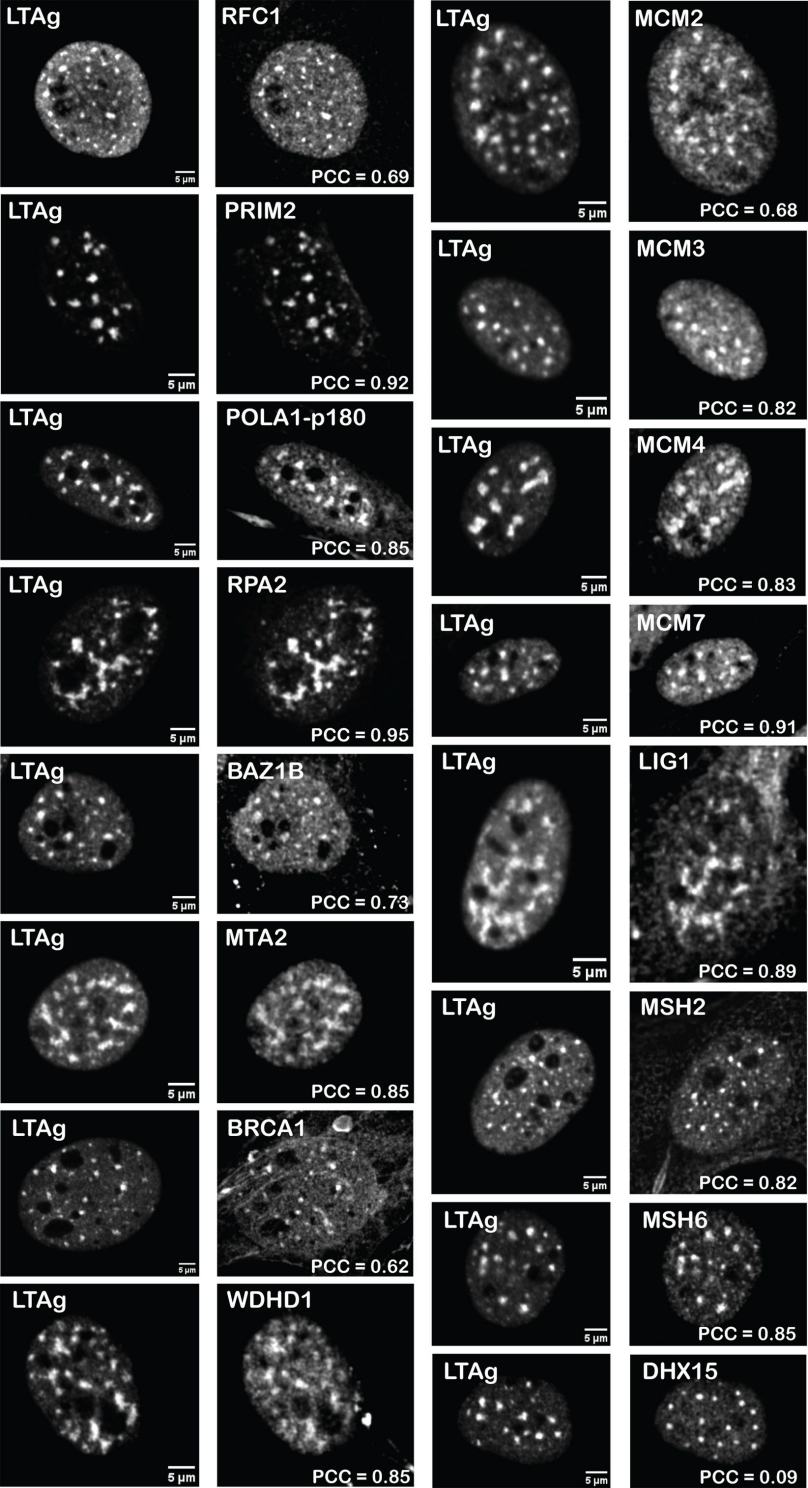
Localization of selected proteins recovered by iPOND and LTAg. C57 MEFs were infected with NG59RA to yield an infection efficiency of 40% and harvested at 28 hours post-infection. LTAg was used as an indicator of VRCs in infected cells. Images shown are representative of cells from 2 to 5 independent experiments. Colocalization was assessed with PCC (Pearson’s colocalization coefficient) as described in Materials and Methods and Fig. S1. PCCs shown correspond to the representative cell in each image.

A subset of the protein candidates was investigated by immunofluorescent analysis. Immunofluorescent staining was used to determine the localization of significantly enriched proteins identified with iPOND within VRCs (as defined by LTAg staining) in MuPyV-infected MEFs. Table 1 shows all proteins meeting the significance threshold from the Pulse and Pulse-Chase data sets, with an LFC over the DMSO control of greater than two. From this set, a subset of candidate VRC components was identified. Candidates were included in the immunofluorescent analysis based on several factors, including the availability of specific primary antibodies, predicted presence within (or absence from) VRCs, and literature supporting a role in the replication of polyomaviruses or other small DNA tumor viruses. RPA2 was added to the immunofluorescence analysis as a positive control, as it has been described as a surrogate marker of VRCs, with high colocalization to LTAg (2, 8). BRCA1 was added to the analysis based on previous research demonstrating BRCA family member involvement in polyomavirus and papillomavirus replication (20–22). To enable co-staining with rat anti-LTAg and downstream colocalization analysis, primary antibodies to mouse nuclear proteins were sought that were produced in rabbits to minimize potential crosstalk and non-specific staining. This condition was a major limiting factor in the selection of candidates, due to a paucity of available antibodies targeting mouse nuclear proteins. Overall, 16 protein targets were selected for validation (Table 1; Fig. 5).

A custom MATLAB script was written to provide a quantitative measure for the extent of colocalization between LTAg and each protein selected from the iPOND list. Infected cells displayed an average nuclear LTAg signal significantly higher than the background. A significant subset of these infected cells demonstrated sharp LTAg foci which delineated VRCs (8). Importantly, nucleoli appeared as marker-poor regions, in cells that were immunostained for either LTAg or selected iPOND candidates (Table S6). Therefore, to avoid an artificially high correlation driven by the presence of nucleoli in the region of interest, the signal correlation was evaluated within an area that did not include nucleoli. We estimated the nuclear fraction occupied by VRCs to discriminate between early-stage infection with more punctate morphologies (1%–10% nucleus area), medium stage with larger viral centers (10%–17% nucleus area as VRC), and late stage with viral tracks (>17%; data not shown). The analysis used a permissive 1% VRC area threshold to capture cells at all stages of infection for analysis.

All proteins evaluated, except for DHX15, demonstrated a high degree of co-localization with LTAg, using Pearson’s Correlation Coefficient to quantify the relationship between both signals within the nucleus (Fig. 5). Calculated PCCs were high both for individual cells (Fig. 5) and populations of cells across multiple independent experiments (Fig. S3). All proteins, except DHX15, demonstrated a PCC with LTAg >0.6, suggesting they colocalize with LTAg and function as part of the MuPyV replisome.

## DISCUSSION

We used iPOND-MS to identify host factors associated with the MuPyV replisome during the early stages of its maturation. Both known and novel PyV genome interactors were recovered on nascent vDNA (Pulse) and vDNA 2 hours post-synthesis (Pulse-Chase). When we compared the Pulse and Pulse-Chase data sets by three different analytic methods, a shift in associated proteins was observed from DNA replication-associated proteins to proteins involved in mRNA processing and nuclear export. Finally, we used immunofluorescence to validate that a subset of newly identified host proteins localized to VRCs. The complete set of proteins identified by iPOND-MS is provided for additional analyses (Tables S1 and S3).

Previous studies identified LTAg as the primary helicase and replication initiator for PyV vDNA (23). Using iPOND, LTAg and other key host replication initiation factors including PCNA, MCM family members, RFC1, RPA, Fen1, topoisomerases, PRIM2, POLA1, and LIG1 were recovered. PCNA and RPA have been shown to localize to MuPyV VRCs using other methods (1, 2, 8, 9). Together, these data support iPOND as a valid method to identify replisome components on vDNA (1, 8). Label-free mass spectrometry used in conjunction with iPOND can generate false positives as well as false negatives (24). Thus, it might be expected that some of the established players in PyV replication would not be captured using this technique, particularly those that do not bind DNA directly. For example, the primary DNA binding domains of RPA are found on the RPA1 subunit, while a single secondary DNA-binding domain is found on RPA2 (25). This association might explain why RPA2 was not significantly enriched in our data set.

Previous work focused on the replisome at pulse and 30-minute pulse-chase timepoints (8). It is likely that the landscape of proteins bound to vDNA changes to accommodate multiple steps in vDNA processing over the course of a 2-hour chase interval. Some members of the MCM complex were not recovered, while MCM2, 3, 4, and 5 were among the most highly enriched proteins in our Pulse data set. This could be due to the different affinity for DNA binding between MCM subunits. Immunofluorescence analysis showed that MCM complex members were highly colocalized with LTAg in punctate VRCs. These data suggest that the MCM complex may play a role in vDNA replication, contrary to previous speculation that the LTAg helicase complex performs all the functions of the MCM complex in unwinding vDNA (23). Further investigation is needed to elucidate the respective roles of LTAg and MCM complex members in the replication of PyV genomes.

The processive DNA polymerase responsible for the duplication of PyV genomes has not been identified conclusively. The current model of the roles of polymerases δ and ε in eukaryotes suggests that, in general, pol ε and pol δ are responsible for leading and lagging strand synthesis, respectively. By contrast, recent data suggest that pol δ can replicate both strands and outcompete pol ε for leading strand synthesis, particularly in the context of DNA damage and helicase-primed DNA (26). In this study, polymerases α and δ were identified by iPOND, but pol ε was not identified. This finding could be a false negative, highlighting the more loose and transient association of pol ε with the replication fork compared to pol δ (26), or might support previous data suggesting that pol ε is not required for polyomavirus replication (27, 28). Zlotkin et al. proposed that pol ε might play a role in repair synthesis rather than replication (27). Our analysis revealed a large complement of host DNA damage sensing and repair proteins are recruited to vDNA, both newly synthesized and at 2 hours post-synthesis. While pol ε was not significantly enriched in either data set, Zlotkin et al. suggested that pol ε could be underrepresented in nascent DNA labeling assays due to a transient association with SV40 DNA. Likewise, we cannot rule out a potential role of pol ε in MuPyV replication.

Certain polyomaviruses interact with components of PML nuclear bodies as part of their early life cycle (29); however, MuPyV replication does not require PML (1). Consistent with this finding, PML nuclear body components, including PML, DAXX, sp100, and SUMO, were not enriched iPOND data sets, supporting the conclusion that MuPyV replication is not dependent on PML.

We identified several proteins that are involved in chromosome reorganization and repair in response to DNA damage, a pathway often hijacked by DNA tumor viruses to facilitate productive infection. Epigenetic regulators, including SMC family proteins, are important for human papillomavirus replication (30). Similarly, we observed several related chromatin modifiers bound to MuPyV genomes. Specifically, SMC family proteins, histone-modifying proteins, including SMC1a, SMCHD1, and HDAC1, were enriched in both data sets, suggesting a role in the organization and modification of MuPyV chromatin.

Viral proteins were detected by iPOND (Fig. 2 and 3), thereby validating the iPOND method. LTAg was identified in both the Pulse and Pulse-Chase data sets, whereas VP1 and VP2 were enriched in the Pulse-Chase data set. We speculate that this observation supports previous work suggesting a role for VP1 and VP2 in binding vDNA directly to regulate viral genome replication and gene expression (31–33).

Comparing the Pulse and Pulse-Chase iPOND data sets reveals differences in the proteome associated with both newly replicated and processed vDNA. These distinctions provide insight into the sequential processing steps PyV genomes undergo prior to packaging. Using 1D GOBP enrichment analysis, several biological processes were identified that significantly differed between the Pulse and Pulse-Chase data sets (Fig. S1). The Pulse data set showed enrichment for DNA repair, while the Pulse-Chase data set showed enrichment for RNA splicing and mRNA processing. Protein translation components were enriched in the Pulse data set, albeit with a small number of proteins identified. Interestingly, proteins associated with mRNA transport, mRNA export out of the nucleus, and nuclear pore organization were all enriched in the Pulse-Chase data set. These observations could suggest several possibilities, including dual DNA- and mRNA-binding functions, which were observed previously with proteins involved in mRNA processing (34). Alternatively, it is possible that vDNA replication and mRNA transcription/processing occur concurrently on RNA-DNA hybrid R-loops, which are captured as crosslinked complexes detected by iPOND (35).

Nuclear pore protein Nup153 has been implicated in the regulation of gene expression through chromatin remodeling *via* interaction with CTCF and cohesin (36). In the Pulse-Chase data set, Nup153 and other nucleoporins were highly enriched, as were regulators of chromatin architecture (Fig. 3; Table S3). Much of the research on polyomavirus genome interaction with the nuclear pore is focused on viral genome entry, rather than mRNA export. Little is known about the host factors mediating sub-nuclear localization of polyomavirus transcription, assembly, or virus exit from the nucleus. Future directions utilizing this data set will include biochemical validation of the interaction of “mature” vDNA with identified host proteins that are critical for these processes. Our study presents a framework for future experiments studying the transition of PyV genomes through replication, the resolution of replication intermediates, and the subsequent transition from replication to transcription. In addition, further studies of the interaction between host factors and maturing PyV genomes should include additional pulse-chase timepoints to elucidate more completely what is likely a highly dynamic and complex process. Overall, the data presented here provide valuable insights into the protein dynamics of MuPyV genomes and act as a resource for future study more precisely elucidating the processing steps that PyV genomes undergo prior to packaging.

## MATERIALS AND METHODS

### Mammalian cell lines and culture

C57 mouse embryonic fibroblasts (MEFs) were obtained from ATCC (SCRC-1008, Manassas, VA). MEFs were incubated at 37°C with 5% CO_2_ in a D10F complete medium consisting of Dulbecco’s Modified Eagle’s Media (DMEM, MilliporeSigma) supplemented with 10% fetal bovine calf serum (FBS, MilliporeSigma), 55 µM β-mercaptoethanol (βME, MilliporeSigma), and 1× antibiotic-antimycotic (Gibco), unless otherwise specified.

### Infections

The wild-type mouse polyomavirus strain NG59RA (37) was used for all infections. Cells were serum starved by incubating overnight in D0F (FBS-free complete medium). Infections were carried out as previously described (8). Briefly, virus supernatant was prepared using sonication and heat (45°C) to liberate virions from cell debris. The virus stock was diluted in adsorption buffer, added to C57 cells, and incubated at 37°C and 5% CO_2_ for the times indicated. The virus stock was diluted such that 40%–60% of cells were LTAg-positive, as measured by immunofluorescence staining against LTAg (9). After infection, cells were grown in complete media with 1% FBS (D1F).

### iPOND

#### Cell infection and EdU incorporation

Cells were grown in 500 cm^2^ cell culture plates (Corning) to 95% confluence and infected as described above. A total of four dishes (~4 × 10^7^ cells/plate) per condition (Pulse or Pulse-Chase) were used for each experiment. At 28 hpi (hours post-infection), media was removed and replaced with D1F media containing 150 µM EdU (+EdU) or DMSO (−EdU) and incubated for 30 minutes. The decision to incubate with EdU for 30 minutes was based on prior research utilizing iPOND to label viral DNA (5), and preliminary optimization studies in polyomavirus infection demonstrating maximal EdU signal in immunofluorescence after 30 minutes of incubation (data not shown). For Pulse conditions, cells were harvested at the end of incubation. For Pulse-Chase conditions, cells were washed quickly two times with D1F, followed by incubation in D1F supplemented with 10 µM thymidine for 2 hours before harvest (14). The decision to conduct the chase incubation for 2 hours was based on previous data demonstrating that viral DNA labeled with EdU travels away from sites of replication to sites of DNA repair progressively over 2 hours (8). To ensure that DNA repair and other processes as distal as possible from sites of replication could be captured, the current study focused on 2 hours pulse-chase.

Upon harvest, cells (~1.6 × 10^8^ total) were processed as described previously (14), with minor modifications. Briefly, the media was removed, and cells were incubated in the presence of 1% formaldehyde (Pierce, 16% stock solution, 28908) diluted in PBS at room temperature (RT) for 20 minutes to crosslink the DNA/protein complexes. The formaldehyde was quenched by the addition of 1.25 M glycine (final concentration 125 µM), and cells were harvested by scraping. Cells were collected by centrifugation at 900 × *g* and washed with PBS three times. Cell pellets were permeabilized at 10^7^ cells/mL in 0.25% Triton X-100/PBS for 30 minutes with rotation at 4°C. Cells were collected by centrifugation as described above and washed once with cold 0.5% BSA/PBS, using the same volume as above. Cells were washed with cold PBS three times and collected by centrifugation as above. For each wash step, the supernatant was poured off to minimize the sample loss. The size of the cell pellet was noted after each step to determine if any loss occurred.

#### EdU labeling using click chemistry

Biotinylation of the permeabilized samples (+EdU or DMSO −EdU control) was performed using Click chemistry, to convert the samples containing EdU-rich DNA into biotinylated DNA. Briefly, the permeabilized cells (10^8^ cells/5 mL) were resuspended in a reaction cocktail containing a final concentration of 10 mM sodium ascorbate, 2 mM CuSO_4_, and 10 µM biotin-azide in PBS with rotation at RT for 2 hours. Cells were washed once in 0.5% BSA/PBS, followed by 1× PBS. Washed cells were resuspended in cold Lysis Buffer [1% SDS/50 mM Tris-HCl, pH 8.0 containing one tablet mini cOmplete protease inhibitor (Roche, 11836153001)] at 1.5 × 10^8^ cells/mL, then sonicated on ice using a Misonix Sonicator 3000 (four-tip microprobe at 20 Watts for a total of 120 seconds, four pulses of 30 seconds at 30-second intervals or until the lysate was clear). Lysates were confirmed to be translucent prior to transfer to a fresh tube. Samples were clarified in a microcentrifuge for 10 minutes at maximum speed and the lysate was filtered through a 100-µm nylon mesh. The lysate was diluted 1:1 with cold 1× PBS/cOmplete protease inhibitor.

#### Streptavidin capture of complexes

Prior to streptavidin capture, 1/20 vol of the biotinylated lysate was removed for DNA analysis (“Input”). An equal volume of 2× SDS-bicarbonate solution was added to the lysate for the capture step. Clarified biotinylated lysates were captured using streptavidin-conjugated magnetic beads (Invitrogen, Dynabeads MyOne Streptavidin T1, 65601). Beads were prepared in 0.5% SDS/PBS/cOmplete protease inhibitor at a 50% bead slurry and added to the lysate at 10^8^ cells per 100 µL beads with an overnight incubation at 4°C with rotation. Proteins bound to the streptavidin beads (either non-specifically or specifically through the biotinylated DNA-protein complexes) were collected using a magnet (Invitrogen, DynaMag−2). The beads were washed with cold lysis buffer, then with 1M sodium chloride/0.1% SDS, and finally twice with cold lysis buffer. At the final wash step, 1/10th volume was removed for DNA isolation and analysis (“Bound”). The remaining beads were collected and resuspended in an equal volume of 1 SDS Buffer (4% SDS/0.125M Tris-HCl, pH 6.8), boiled for 30 minutes, and cleared by removing the beads in the magnet. The clarified supernatant was transferred to fresh tubes for either western blot analysis or mass spectrometry analysis (see below).

### DNA isolation and qPCR analysis of iPOND samples

#### DNA isolation

The “Bound” sample beads were removed *via* magnet prior to DNA extraction. Samples were extracted with an equal volume of phenol:chloroform:isoamyl alcohol (25:24:1) and the aqueous phase was transferred to a new tube. The phenol phase was re-extracted with an equal volume of 1X TE (100 mM Tris, pH 8.0/10 mM EDTA). The aqueous phases were combined and extracted with an equal volume of chloroform:isoamyl alcohol (24:1). Sodium acetate (3M, 1/10 vol) was added to the lysates prior to further purification using the QIAquick PCR Purification kit (QIAGEN, 28104) according to the manufacturer’s protocol. The DNA was eluted from the column in 30–50 µL 10 mM Tris, pH 7.2. The DNA concentration was determined using the Qubit dsDNA HS kit (Invitrogen, Q33230) and Qubit 4 Fluorometer (Invitrogen, Q33238).

#### qPCR quantitation of viral DNA

The presence of vDNA was confirmed with qPCR using primers to the VP1 region (5′TGGGAGGCAGTCTCAGTGAAA3′; 5′TGAACCCATGCACATCTAACAGT3′). Quantification of the vDNA was performed using a serial dilution of pUC18-NG59RA plasmid DNA as a standard on a BioRad CFX96 Real-time PCR Detection System Absolute. The number of vDNA genomes was determined for “Input” and “Bound” samples. The “Bound” samples obtained by processing EdU-free cells (DMSO control) did not contain detectable vDNA.

### Immunoblot analysis

“Input” and “Bound” samples were mixed with an equal volume of 2X SDS sample buffer (8% SDS/0.25M Tris-HCl, pH 6.8/10% 2-mercaptoethanol/10% glycerol/0.005% bromophenol blue) and boiled for 10 minutes prior to loading. “Bound” sample aliquots were removed and submitted for mass spectrometry. Boiled samples were resolved by SDS-PAGE using 4%–20% polyacrylamide gels (BioRad, 5671094) and transferred to PVDF membrane in 1 tris-glycine/20% methanol at 4°C for 2 hours. Membranes were blocked with 5%milk/TBST (20 mM Tris-HCl, pH 7.4/150 mM NaCl/0.1% Tween 20) at RT and incubated with primary antibodies diluted in 5% BSA/TBST [anti-TAg (1:100, PN116, gift of B. Schaffhausen); anti-PCNA (1:2500, Cell Signaling, or 1:2,000, Novus, PC10)] overnight at 4°C. Horseradish peroxidase-conjugated secondary antibodies were diluted in 5% milk/TBST and incubated with membranes at RT. Signals were detected using enhanced chemiluminescence (Pierce, 34580) per the manufacturer’s protocol and imaged using an ImageQuant LAS 4000 (GE Healthcare). In the “Input” lanes, 0.5% of the total lysate was loaded, while in the “Bound” lanes, 5% of the total lysate was loaded.

### Mass spectrometry of iPOND samples

#### Sample preparation for mass spectrometry

iPOND samples and lysate input were denatured in 5% (wt/vol) SDS, 50 mM Tris-HCl, pH 8.5, 10 mM Tris(2-carboxyethylphosphine) (TCEP) and 40 mM chloroacetamide by boiling at 95°C for 10 minutes. Each sample was digested using the SP3 method (38). Carboxylate-functionalized SpeedBeads (GE Life Sciences) were added to the lysates. The addition of acetonitrile to 80% (vol/vol) was used to bind the proteins to the beads. The beads were washed twice with 80% (vol/vol) ethanol and twice with 100% acetonitrile. Proteins were digested with Lys-C/Trypsin (Promega) overnight at 37°C. Acetonitrile was added to 95% (vol/vol) to bind the tryptic peptides back to the SpeedBeads followed by one wash with 100% acetonitrile. Peptides were eluted from the SpeedBeads with 1% (vol/vol) trifluoroacetic acid in 3% (vol/vol) acetonitrile, dried by vacuum centrifugation, and stored at −20°C until analysis. The lysate input sample was fractionated with high pH reversed-phase C18 UPLC using a 0.5 mm × 200 mm custom-packed UChrom C18 1.8 µm 120 Å (Nano LC/MS) column with mobile phases 10 mM ammonium formate, pH10 in water and acetonitrile. Peptides were gradient eluted at 20 µL/minute from 2% to 50% acetonitrile in 50 minutes concatenating for a total of 12 fractions using a Waters M-class UPLC (Waters). Peptide fractions were then lyophilized by vacuum centrifugation and stored at −20°C until analysis.

#### Mass spectrometry analysis

Samples were suspended in 3% (vol/vol) acetonitrile, a 0.1% (vol/vol) trifluoroacetic acid and directly injected into a C18 1.7 µm, 130 Å, 75 µm × 250 mm M-class column (Waters), using either a Thermo Ultimate 3000 RSLC nano or a Waters M-class UPLC. Peptides were eluted at 300 nL/minute using a gradient from 2% to 20% acetonitrile over 100 minutes into either a Q-Exactive HF-X or an Orbitrap Fusion mass spectrometer (both Thermo Scientific). Precursor mass spectra (MS1) were acquired at a resolution of 120,000 from 350 to 1550 m/z with an automatic gain control (AGC) target of 3E6 and a maximum injection time of 50 ms (HF-X) or 380 to 1,500 m/z with an AGC of 2E5 and a maximum injection time of 50 ms (Fusion). Precursor peptide ion isolation width for MS2 fragment scans was 1.4 m/z, and the top 12 most intense ions were sequenced (HF-X) or 1.6 m/z at Top Speed for 3 seconds (Fusion). All MS2 spectra were acquired at a resolution of 15,000 (HF-X) or in the linear ion trap (Fusion) with higher energy collision dissociation (HCD) at 27% normalized collision energy (HF-X) or 35% collision energy (Fusion). An AGC target of 1E5 and 100 ms maximum injection time was used (HF-X) or 1E4 and 35 ms (Fusion) maximum injection time. Raw files were searched against the UniProt (39) *Mus musculus* databases UP000000589 using MaxQuant (16) version 1.6.3.4 with cysteine carbamidomethylation as a fixed modification. Methionine oxidation and protein N-terminal acetylation were searched as variable modifications. All peptides and proteins were thresholded at a 1% false discovery rate (FDR). LFQ and iBAQ intensities were cyclic loess normalized and log2 fold changes and *P*-values were calculated using the LIMMA package (15) in Bioconductor (40, 41) using a custom R-script.

### Data analysis

Proteomics data processing and analysis were performed using MaxQuant Perseus (42). To enable comparisons between the EdU and DMSO sample groups, identified proteins were filtered to retain only those that had valid intensity values in at least three biological replicates in either the EdU or DMSO groups. Enrichment was defined as an LFC for EdU/DMSO of greater than two and adjusted *P* < 0.05, as calculated using the custom LIMMA R-script. For comparison of Pulse versus Pulse-Chase samples, proteins were similarly filtered to retain only those that had valid intensity values in at least three biological replicates in both groups, as calculated using the custom LIMMA R-script. Enrichment was defined as an LFC greater than two and *P* < 0.05. 1D annotation enrichment analysis was performed using MaxQuant Perseus (43). More information on data processing and analysis workflows in Perseus can be found in the literature cited here, as well as on the MaxQuant YouTube channel at https://www.youtube.com/@MaxQuantChannel.

### STRING analysis

Selected proteins were searched in the STRING network web interface (44) (v12) using the following settings: *Mus musculus* (organism); full STRING network (network type); confidence (meaning of network edges); text mining, experiments, databases (active interaction sources); medium confidence 0.4 (minimum required interaction score); and none/query proteins only (interactors to show). MCL clustering was used, with an inflation parameter of three, and dotted lines denoting the identified edges between clusters.

### Immunofluorescence microscopy

C57 MEFs were seeded in 96-well PerkinElmer Phenoplates (P/N 6055300) at a density of 5,300 cells/cm^2^ in complete media, then infected after 24 hours as described above. All media changes and washes were performed by gently pipetting the liquid, always leaving at least 25–50 µL liquid in the well, to minimize cell loss and nuclear deformation. The list of antibodies and dilutions used is provided in Table S6. At 28 hpi, growth media was removed, cells were washed once in PBS, and then placed on ice for 8 minutes. All subsequent washes and incubations were carried out on ice with cold reagents and very gentle rocking (1 rpm) unless otherwise noted. Cells were fixed in 4% paraformaldehyde (PFA) without rocking, then permeabilized in blocking buffer (10% bovine calf serum/PBS) with 0.05% Triton X-100 for 20 minutes on ice without rocking. Cells were incubated in blocking buffer overnight at 4°C, then incubated with primary antibody (diluted in PBS supplemented with 1% BSA and 0.2% Triton X-100) overnight at 4°C, followed by incubation with Alexa fluor-conjugated secondary antibody (diluted 1:2500 in PBS freshly supplemented with 0.05% Tween 20) for 90 minutes at RT. Washes were performed using primary or secondary antibody dilution buffer three times for a total of approximately 5 minutes. Cells were stained with 1.6 µM Hoechst in PBS for 10 minutes at RT, then washed once with PBS prior to imaging.

Images were acquired on a PerkinElmer Opera Phenix High Content Screening system, using a 1.1 NA 40× water immersion objective (one binning). Hoechst nuclear stain was visualized at 375 nm excitation, 435–480 nm emission, AF488 at 488 nm excitation, 500–550 nm emission, AF568 at 561 nm excitation, 570–630 nm emission, and AF647 at 640 nm excitation, 650–760 nm emission. The absence of signal bleed-through was confirmed using cells stained with single fluor and imaged in the other three channels (data not shown). Z-stacks were acquired, and the best plane for the cells of interest was identified using Fiji software (45). The “ROI manager” function in Fiji was used to produce the image of single nuclei of interest at the sharpest z-plane (arbitrary visual determination). The output image pair was processed through MATLAB as described below.

### MATLAB analysis

A custom MATLAB (Mathworks) script was generated to correlate nuclear signals in two different channels [(LTAg) and (iPOND candidate)] using the Pearson Correlation Coefficient (PCC) over a region that excludes nucleoli (and other areas where the LTAg signal is dim), in a subset of cells which display significant nuclear fraction of VRCs (“VRC_area_fraction” greater than 1%). The script automatically delineates both VRCs and nucleoli using the cell-dependent median nuclear LTAg signal “median_LTAg.” Nucleoli were successfully defined as regions where the LTAg signal is lower than 0.6× median_LTAg, while VRCs were defined as regions where the LTAg signal is greater than two times median_LTAg. As shown in Fig. S2, for each nucleus detected in channel 2, the MATLAB script: (i) outputs a figure that delineates VRCs, nucleoli, and the PCC_area (figure used to tune key parameters and quality check before recording numerical outputs); (ii) estimates the VRC area fraction in the nucleus; and (iii) in VRC-positive cells, calculates the PCC value over the PCC area (shown in the figure bottom panels). If the VRCs were too small or absent, the script output was “NaN” (not a number). NaN values were extracted to restrict PCC analysis to VRC-positive cells.

## Data Availability

The mass spectrometry proteomics data have been deposited to the ProteomeXchange Consortium via the PRIDE (46) partner repository with the data set identifier PXD055210 and 10.6019/PXD055210. MATLAB code is publicly available at https://github.com/UCBoulder/Garcea_Rep1

## AFTER EPUB

*[This article was published on 31 October 2024 with an error in Fig. 4. The figure was updated in the current version, posted on 19 November 2024.]*

## SUPPLEMENTAL MATERIAL

Figure S1 jvi.00790-24-s0001.eps iPOND dataset biological process enrichment.

Figure S2 jvi.00790-24-s0002.eps MATLAB analysis pipeline to determine colocalization between LTAg and iPOND candidates.

Fig. S3 jvi.00790-24-s0003.tif Colocalization of selected proteins identified by iPOND with MuPyV LTAg.

Supplemental legends jvi.00790-24-s0004.docx Legends for Fig. S1 to S3.

Table S1 jvi.00790-24-s0005.xlsx EdU vs DMSO Limma output.

Table S2 jvi.00790-24-s0006.xlsx Limma output for proteins with no DMSO intensity.

Table S3 jvi.00790-24-s0007.xlsx Pulse vs Pulse-Chase Limma output.

Table S4 jvi.00790-24-s0008.xlsx Limma output for proteins with no EdU intensity in Pulse or Pulse-Chase.

Table S5 jvi.00790-24-s0009.xlsx iPOND dataset 1D KEGG name enrichment.

Table S6 jvi.00790-24-s0010.docx Antibodies used for immunofluorescence experiments shown in Fig. 4, Table 1, and Fig. S3.

ASM does not own the copyrights to Supplemental Material that may be linked to, or accessed through, an article. The authors have granted ASM a non-exclusive, world-wide license to publish the Supplemental Material files. Please contact the corresponding author directly for reuse.
