# Supplementary figures and images for: Proteome profiling of polyomavirus nuclear replication centers using iPOND
Source: J Virol. 2024 Oct 31;98(11):e00790-24. doi: 10.1128/jvi.00790-24 (PMC11575236; doi:10.1128/jvi.00790-24)

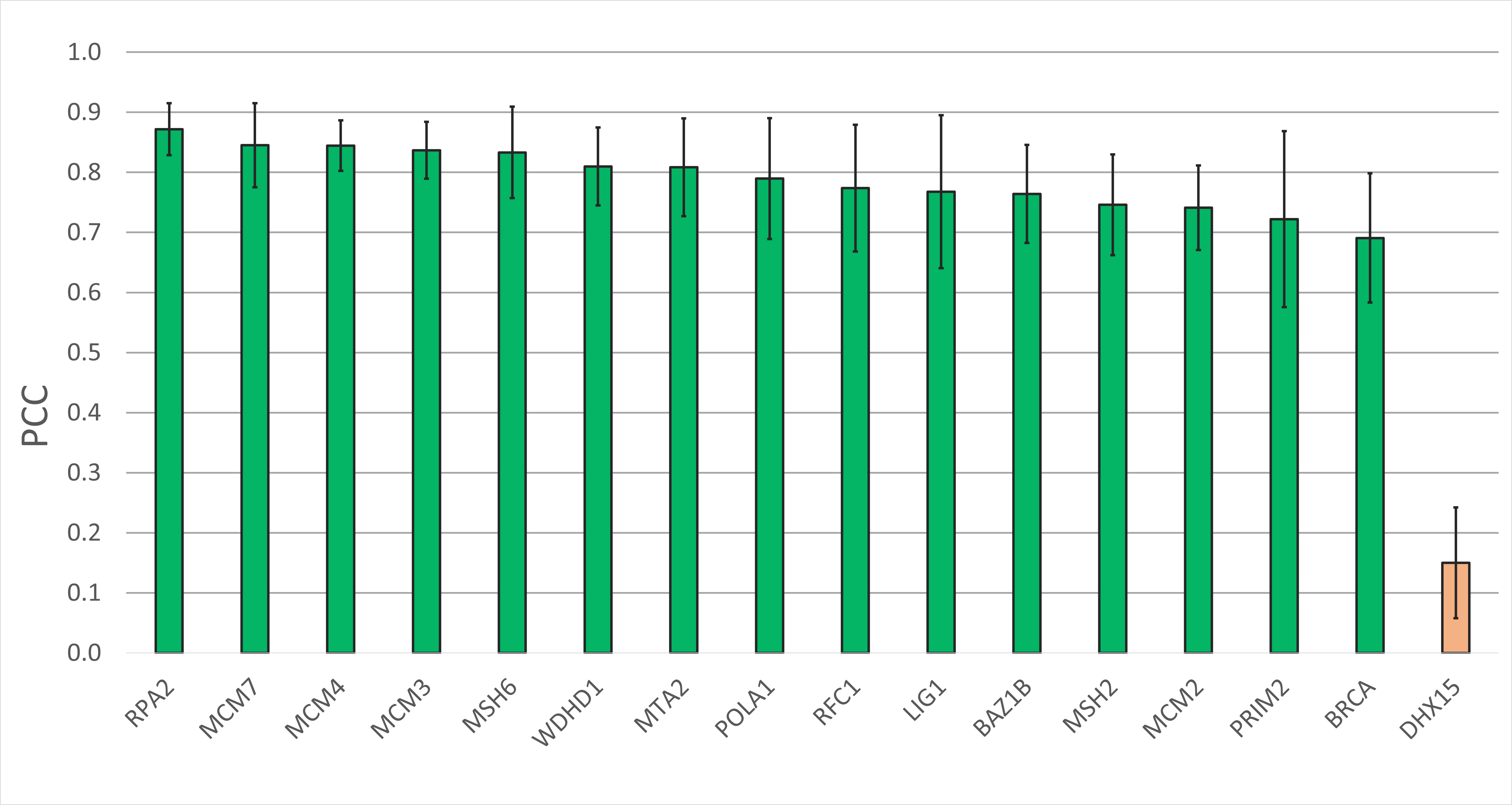

Supplement: Fig. S3 — Colocalization of selected proteins identified by iPOND with MuPyV LTAg. [file jvi.00790-24-s0003.tif]
