# Supplementary material for: Proteome profiling of polyomavirus nuclear replication centers using iPOND
Source: J Virol. 2024 Oct 31;98(11):e00790-24. doi: 10.1128/jvi.00790-24 (PMC11575236; doi:10.1128/jvi.00790-24)
Supplement: Supplemental legends — Legends for Fig. S1 to S3. [file jvi.00790-24-s0004.docx]

**Supplemental Figure Legends**

**Fig S1. iPOND dataset biological process enrichment.** Scatter plot of 1D enrichment analysis of the association between the log2 fold change in intensity between proteins detected in the Pulse-Chase and Pulse conditions and Gene Ontology Biological Process (GOBP) identifiers. The enrichment score (x-axis) is plotted against the significance (y-axis). The size of each bubble corresponds to the number of identified proteins belonging to each GOBP group.

**Fig S2. MATLAB analysis pipeline to determine colocalization between LTAg and iPOND candidates.** Example MATLAB analysis of an infected MEF nucleus displaying significant VRCs. The cell was stained with Hoechst to visualize nuclei (ch1, not shown), anti-LTAg (ch2, (A, B, C)) and anti-MSH2 (ch3, (D)) as described in Methods. The script outputs an opaque blue overlay of the VRCs (A), an opaque blue overlay of the nucleoli (B), transparent green (C) and red (D) overlays of the area to be analyzed for colocalization from each channel, excluding the label-poor nucleoli. These outputs permit manual qualitative control as well as parameters for tuning prior to writing a PCC output value.

**Fig S3.** **Colocalization of selected proteins identified by iPOND with MuPyV LTAg.** WT MEFs were infected with NG59RA at an MOI of approximately 0.5 and harvested at 28 hours post infection. Immunofluorescence was performed as described in Methods, with LTAg as a marker of VRCs. Colocalization was assessed with PCC (Pearson’s colocalization coefficient) as described in Methods and Fig S1. n = 6-11 cells across 2-3 independent experiments.
