# Supplementary material for: Proteome profiling of polyomavirus nuclear replication centers using iPOND
Source: J Virol. 2024 Oct 31;98(11):e00790-24. doi: 10.1128/jvi.00790-24 (PMC11575236; doi:10.1128/jvi.00790-24)
Supplement: Table S6 — Antibodies used for immunofluorescence experiments shown in Fig. 4, Table 1, and Fig. S3. [file jvi.00790-24-s0010.docx]

**Table S6.** Antibodies used for immunofluorescence experiments shown in Fig 4, Table 1 and Fig S3.

| **Protein Target** | **Manufacturer** | **Catalog #** | **Dilution** |
| --- | --- | --- | --- |
| LTAg | Santa Cruz | SC-54379 | 1:200 |
| LIG1 | Abclonal | A1858 | 1:100 |
| BAZ1B/WSTF | ThermoFisher | PA5-121245 | 1:100 |
| MCM2 | CST | CST 4007 | 1:100 |
| MCM3 | CST | CST 4012 | 1:100 |
| MCM4 | ThermoFisher | PA5-29039 | 1:100 |
| MCM7 | ThermoFisher | 11225-1-AP | 1:100 |
| MTA2 | NOVUS | NBP1-82615 | 1:100 |
| RFC1 | NOVUS | NBP2-56364 | 1:100 |
| p180 | ThermoFisher | BS-6166R | 1:100 |
| MSH2 | ThermoFisher | PA5-79695 | 1:100 |
| MSH6 | ThermoFisher | MA5-32676 | 1:100 |
| MSH2 | CST | CST2017 | 1:100 |
| DHX15 | NOVUS | NBP2-13919 | 1:100 |
| RPA2 | ThermoFisher | PA5-110204 | 1:100 |
| PRIM2 | ThermoFisher | 11788-1-AP | 1:100 |
| Polα1/ p180 | Kind gift of Dr. Takeshi Mizuno | | 1:50 |
| BRCA1 | SCBT | sc-135732 | 1:100 |
| AND1/WDHD1 | ThermoFisher | PA5-51517 | 1:100 |
